# CBCT in Evaluation of Root Canal Preparation—A Scoping Review

**DOI:** 10.3390/dj14020114

**Published:** 2026-02-14

**Authors:** Andreia Vidal, Ana Moura Teles, Miguel Cardoso, Maria Bartolomeu, Rita Noites

**Affiliations:** 1 Universidade Católica Portuguesa, Faculty of Dental Medicine, 3504-505 Viseu, Portugal; s-avidal@ucp.pt (A.V.);; 2 Universidade Católica Portuguesa, Faculty of Dental Medicine, Centro de Investigação Interdisciplinar em Saúde (CIIS), 3504-505 Viseu, Portugal; mabcardoso@ucp.pt (M.C.); mbartolomeu@ucp.pt (M.B.)

**Keywords:** CBCT, endodontics, dental instruments, root canal preparation, root canal therapy

## Abstract

Cone-beam computed tomography (CBCT) is widely utilized in endodontics for evaluating root canal shaping outcomes, offering critical three-dimensional imaging capabilities. This study aims to assess the differences in apical and root canal preparation across various instrumentation techniques using CBCT. A systematic search of the Medline database (via PubMed) and Web of Science was performed up to 12 April 2025, yielding a total of 70 studies, with 45 full-text articles assessed for eligibility; 28 were included in the review. Studies showed great heterogeneity in experimental design, anatomical variables, and outcome measurements. The results indicate that rotary instruments, such as ProTaper Next^®^ and XP-Endo Shaper^®^, were reported more frequently or showed favorable shaping trends in individual studies. Although rotary systems often appeared advantageous, conclusions were limited by study design variability and a lack of correlation with clinical outcomes. The evidence highlights the need for standardized methodologies and further research, especially on manual techniques. CBCT remains a valuable research tool despite inherent spatial resolution limitations.

## 1. Introduction

The primary objective of endodontic treatment is to eliminate microorganisms that invade the pulp chambers and root canals, thereby aiding in the preservation of natural teeth [1,2,3]. This treatment protocol includes several critical stages: mechanical preparation and disinfection, root canal preparation, and obturation. During these stages, the root canal is sealed with specific materials to restore the anatomical integrity of the tooth [4]. To achieve optimal outcomes in endodontic procedures, it is essential to ensure meticulous preparation and cleaning, as well as effective disinfection of the root canal.

Root canal preparation plays a crucial role in facilitating the obturation process and enhancing treatment success [5,6]. This phase involves the use of endodontic instruments, commonly known as files, to enlarge the root canals, maintaining their original shape and trajectory [7,8]. Ideally, a tapered morphology is achieved, narrowing towards the apex in both straight/wide and curved/narrow canal configurations [7]. Sodium hypochlorite between each procedural step is vital for the effective elimination of microorganisms and debris [9,10,11,12,13]. Root canal preparation techniques have evolved significantly due to technological advancements and can be performed either manually or mechanically. Manual instrumentation, developed by Edward Maryard, uses stainless steel files, while rotary techniques utilize Nickel-Titanium (Ni-Ti) rotary instruments, enabling faster and more efficient root canal preparation. Systems such as ProTaper Universal and XP-Endo Shaper provide flexibility and better adaptation to canal morphology. Multiple techniques have been explored to enhance quality and reduce operator fatigue: these include the conventional, balanced force, step-back, flared, rotary Ni-Ti, and crown-down techniques [1,4,7,14,15,16].

Cone Beam Computed Tomography (CBCT) has emerged as a sophisticated radiological modality, using cone-shaped X-rays to produce high-resolution images with reduced radiation exposure [17,18,19]. In endodontics, CBCT is instrumental for diagnosing root canal morphology, periapical bone loss, fractures, and resorptions, particularly where standard radiographs are inadequate [20,21,22]. Despite its benefits, CBCT should be considered a complementary rather than a replacement tool for routine digital radiography [23].

Given the rapidly growing and heterogeneous literature on the use of CBCT for evaluating root canal shaping, a scoping review is warranted to systematically map the characteristics, scope, and gaps in current research. This scoping review aims to provide an evidence map of ex vivo studies that have used CBCT to assess root canal and apical preparation with various instrumentation techniques, highlighting trends, evidence clusters, and areas requiring further investigation.

## 2. Materials and Methods

### 2.1. Protocol and Registration

This scoping review was conducted and reported in accordance with the Preferred Reporting Items for Systematic Reviews and Meta-analyses for Scoping Reviews (PRISMA-ScR). All procedures, including study selection and data charting, were performed independently by two reviewers; any disagreements between the reviewers were resolved through consensus or, if necessary, by a third reviewer.

### 2.2. Databases and Search Strategy

A comprehensive literature search was conducted across the databases Medline (via PubMed) and Web of Science (WoS). Search terms incorporated synonyms and free-text terms related to CBCT and root canal preparation. The search question was developed according to the PCC (Population, Concept, Context) framework, in alignment with Scoping Review methodology: Population, extracted teeth (ex vivo studies); Concept, assessment of root canal preparation using CBCT; Context, various instrumentation techniques.

The search strategy for PubMed was: (((CBCT) OR (cone-beam computed tomography)) AND ((“root canal preparation” OR “root canal instrumentation”) OR “mechanical preparation”)) AND ((“cleaning efficiency” OR “shaping ability”) OR “outcome”).

A similar strategy was used for Web of Science: (((CBCT) OR (cone-beam computed tomography)) AND (((“root canal preparation”) OR (“root canal instrumentation”)) OR (“mechanical preparation”))) AND (((“cleaning efficiency”) OR (“shaping ability”)) OR (outcome)).

### 2.3. Study Selection

Titles and abstracts were screened independently by two reviewers. Full-text articles were obtained for potentially relevant studies. Eligibility was determined against the inclusion criteria, with reasons for exclusion recorded, and discrepancies were resolved by discussion or a third reviewer.

### 2.4. Inclusion and Exclusion Criteria

The inclusion criteria for this review comprised studies published in English between 2014 and 2024 that provided full-text availability and were conducted as ex vivo studies. The exclusion criteria encompassed non-English articles, literature reviews, editorials, or conference abstracts, and studies focusing on deciduous teeth or artificial tooth replicas.

### 2.5. Data Charting and Synthesis

Data were charted using a standardized form developed by the team. The following variables were extracted: year, country, tooth type, experimental design, file system, outcome measures, measurement formulas, and main findings. The results were mapped and synthesized descriptively, focusing on the distribution of instrumentation systems, CBCT approaches, and areas of evidence concentration or gaps.

## 3. Results

**Figure 1:**
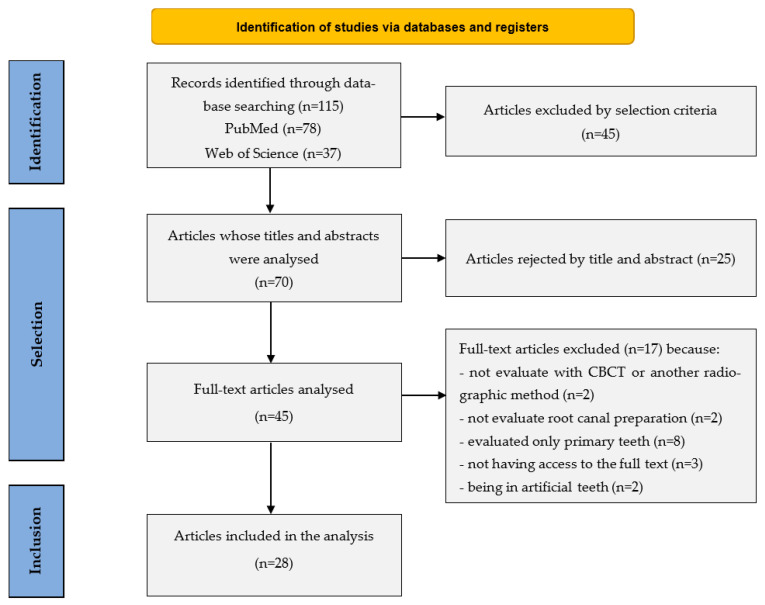
Flowchart of PRISMA-ScR for this scoping review.

This scoping review identified a total of 28 studies meeting the inclusion criteria after screening 70 full texts, following removal of duplicates and title/abstract screening (Figure 1). Included studies were mapped for characteristics including instrumentation techniques, file types, tooth types, and CBCT-based analysis (Table 1). The broad heterogeneity in study design and outcome measures highlights the variability in current research, with a predominance of rotary instrumentation systems.

**Figure 2:**
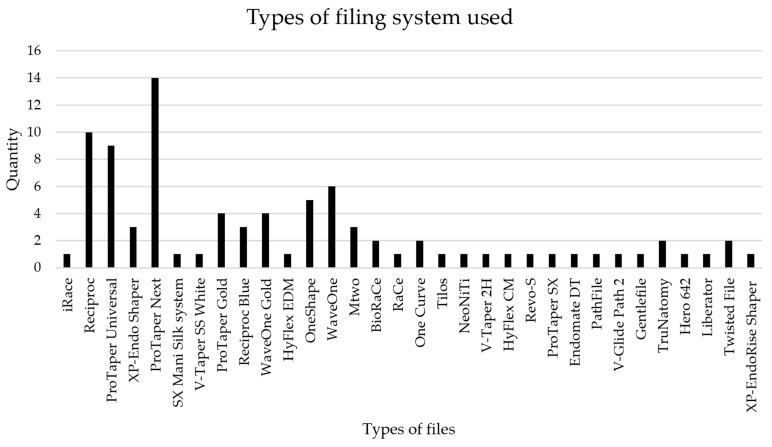
Relationship of the number of file systems present in the articles examined.

Most studies focused on rotary instrumentation systems, with ProTaper Next^®^ being the most extensively evaluated (featuring in 14 articles), followed by Reciproc^®^ (10 articles) and ProTaper Universal^®^ (9 articles) (Table 1, Figure 2). Other systems were investigated less frequently, indicating research concentration on a few established rotary systems: the WaveOne^®^ system was assessed in 6 studies, while the OneShape^®^ system was assessed in 5 studies. ProTaper Universal Gold^®^ and WaveOne Gold^®^ were examined in 4 studies each. BioRaCe^®^, One Curve^®^, TruNatomy^®^, and Twisted File^®^ were each the subject of 2 investigations, with other systems being referenced in only 1 article. While no pooled effect sizes are reported given heterogeneity, individual studies frequently report performance metrics such as canal transportation and centering ratios, suggesting rotary files generally maintain canal anatomy more effectively than manual systems. However, based on the data presented in Figure 2, it can be concluded that most systems were analyzed in a single study, highlighting a pressing need for further investigation into their efficacy in root canal preparation.

### 3.1. Teeth Typology

**Figure 3:**
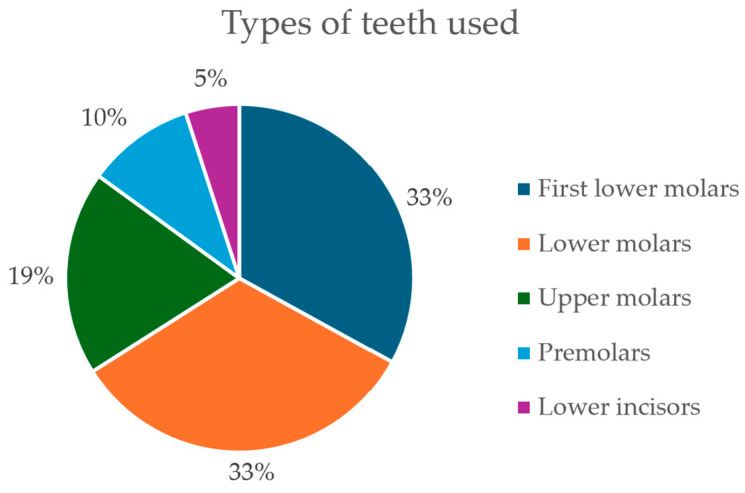
Distribution of the type of teeth across studies.

The majority of studies examined molars, mainly first lower molars [25,30,31,33,41,45,49], followed by both lower molars [27,28,34,38,40,42,47], and upper molars [24,37,43,46], premolars [39,44], and lower incisors [26] (Figure 3). The majority of permanent teeth examined, encompassing both single-rooted and multi-rooted varieties, were extracted due to periodontal issues [24,26,27,32,36,45], the presence of carious lesions [27], prosthetic complications [32], or the degree of curvature of the roots [24,25,28,29,30,31,32,33,34,35,37,38,39,41,43,46,48]. The type of tooth and the number of root canals are variables that influence the results; an increased number of root canals necessitates more meticulous procedures to achieve optimal outcomes.

The anatomical variability and number of root canals across tooth types influenced results, requiring more meticulous shaping in multi-rooted teeth. Subgrouping by tooth type reveals consistent attention to the complexities of curved and narrow canals, although direct quantitative comparison is limited by study design differences.

### 3.2. Manual Versus Rotary Systems

Only a limited number of studies directly compared manual and rotary instrumentation [44]. The discrepancies in conclusions across various studies may stem from the examination of multiple files and preparation techniques, leading to divergent findings and relationships [30,31,32,33,34,35,36,37,38,39,40,41,42,51].

### 3.3. Outcome Measures and Calculation Methods

With respect to file performance in terms of transportation and centralization, several studies reported no significant differences that would affect root canal geometry [24,30,37,41,42,44,47]. However, certain files, such as ProTaper Universal^®^ [25], SX Mani Silk^®^ [35], and Gentlefile^®^ [38], exhibited greater transportation, while others, including ProTaper Gold^®^ [52] and XP-Endo Shaper^®^ [36], demonstrated reduced transportation, thereby enhancing instrumentation. In relation to centralization, files such as Revo-S^®^ [31], Patch File^®^ [34], and XP-Endo Shaper^®^ [36,51] displayed superior root canal centralization compared to the predetermined ideal.

Ideally, minimal transportation and improved centralization contribute to more efficient outcomes, thereby reducing treatment duration and associated complications. All of these factors are essential for achieving an optimal root canal shape, which facilitates effective root canal cleaning. It is noteworthy that the studies in question came to varying conclusions.

Although some investigations identified specific files, such as XP-Endo Shaper^®^ [25,36] and V-Taper^®^ [28], as exceptional, others found no significant differences among files, resulting in the absence of a definitive superior option [24,26,30,37,39,40,41,43,44,47]. Overall, the studies suggest that the files tested are suitable for endodontic treatment without inflicting significant issues on root canal shape or cleaning; however, one article indicated that no file excelled in both transportation and centralization [42].

The pre- and post-instrumentation CBCT images were utilized to assess the outcomes and identify any changes. In addition to image analysis, calculations were conducted to ascertain file centralization and transportation. The majority of articles employed the formulas depicted in Figure 4 to calculate file centralization and transportation using CBCT images [24,25,26,27,28,29,30,31,32,33,34,35,36,38,39,40,41,42,43,44,45,47].

In these equations, *m*1 and *m*2 denote the shortest distance from the mesial surface to the periphery of the root canal, measured before and after instrumentation, respectively. Meanwhile, *d*1 and *d*2 represent the corresponding measurements for the distal surface. Pagliosa et al. [37] employed a formula proposed by Loizides et al., which encompasses the following metrics:

- Root canal transportation (CT) is calculated as CT = MT − DT,
- Root canal centering (CA) is expressed as CA = (*m* total − *d* total)/CD

In these equations, MT and DT signify the transportation distances for the mesial and distal surfaces, respectively, while CD denotes the diameter of the root canal. All of these criteria were determined based on the average values obtained from each root canal (total *m* and total *d*).

## 4. Discussion

Endodontic treatment aims to preserve the natural teeth and involves multiple phases that can be time-consuming and technically demanding. A growing range of instrumentation techniques has been developed to improve efficiency and safety, and a clear understanding of their impact on canal transportation and centralization is essential for informed clinical decision-making [1,3,7]. This scoping review mapped ex vivo CBCT-based studies evaluating root canal preparation with different instrumentation systems, focusing on how current research is distributed across file types, tooth anatomy, and assessment methods [24,25,26,27,28,29,30,31,32,33,34,35,36,37,38,39,40,41,42,43,44,45,46,47,48,49,50,52].

Manual instrumentation, although historically established and capable of producing acceptable outcomes [44], is under-represented in the available literature when compared with rotary techniques. Rotary and reciprocating systems dominate current research, with ProTaper Next^®^, ProTaper Universal^®^, and ProTaper Gold^®^ among the most frequently investigated file systems, particularly in lower molars. Many studies also incorporated additional parameters such as root canal curvature, dentin removal, and change in canal volume or cross-sectional area, which illustrates the methodological breadth but also contributes to heterogeneity in reported outcomes [46,50,51].

CBCT has become an important imaging tool in endodontics, providing three-dimensional visualization for diagnosis, treatment planning, and pre- and post-instrumentation assessment [18,30,35,40,52,53]. It offers clinically acceptable image quality at relatively low radiation doses and enables non-destructive evaluation of canal geometry before and after preparation. However, its spatial resolution remains a key limitation in the ex vivo research context. Typical clinical CBCT voxel sizes (approximately 75–100 µm) are substantially larger than those achievable with micro-computed tomography, which can reach sub-20 µm resolutions. This limits the precision with which subtle changes in canal morphology, such as small degrees of transportation or centralization, can be detected or quantified. Consequently, CBCT is suitable for comparative, exploratory assessment of shaping outcomes but may not be sensitive enough for definitive evaluation of fine morphological differences, for which micro-CT remains the reference standard in ex vivo research. Interpretations of detailed shaping performance in the included studies should therefore be made with this constraint in mind.

Most included studies assessed shaping outcomes using transportation and centering ratios, frequently calculated with formulas derived from Gambill and colleagues [52], while some used alternative approaches [37]. Despite differences in specific equations and measurement protocols, the general conceptual focus was consistent: quantifying how well each system maintained the original canal trajectory and minimized unwanted deviation. Several systems, such as Revo-S^®^ [31], Patch File^®^ [34], and XP-Endo Shaper^®^ [36,51], were reported to perform favorably in terms of centering, whereas ProTaper Gold^®^ [52], Reciproc Blue^®^ [52], and other modern systems often showed relatively low transportation in the contexts studied. However, given the heterogeneity across studies in tooth type, curvature, operator protocols, and imaging parameters, these findings should be interpreted as indications of trends within specific experimental conditions, rather than as evidence of clear superiority between systems.

Nonetheless, the review had limitations, including the disparity in the volume of studies comparing manual and rotary methods, variations in assessment techniques, and insufficient research concerning specific files and types of teeth. Substantial variability in experimental design, canal morphology, instrumentation protocols, and CBCT acquisition parameters further reduces the comparability of results and precludes reliable estimation or pooling of effect sizes. In addition, the included studies were ex vivo, which restricts the extrapolation of findings to clinical outcomes such as healing, pain, or long-term tooth survival.

Within these constraints, this scoping review highlights clear areas where evidence is clustered, particularly around a small number of popular rotary systems in molar teeth, as well as substantial gaps in the literature. Future research would benefit from more balanced exploration of manual and rotary techniques, broader inclusion of different tooth types and anatomies, and greater standardization of outcome measures and imaging protocols. The integration of higher-resolution modalities, such as micro-CT, in ex vivo work, combined with well-designed clinical studies, will be essential to translate shaping performance metrics into meaningful clinical recommendations. Overall, the findings reinforce the importance of careful instrument selection and protocol design while underscoring the need for more rigorous and standardized research to support evidence-based endodontic practice.

## 5. Conclusions

The objective of this study was to map, using CBCT, how different instrumentation techniques have been used to assess apical and root canal preparation in ex vivo models. This scoping review identified a heterogeneous body of evidence, with substantial emphasis on rotary systems and a comparatively smaller number of studies addressing manual techniques. The included studies most frequently evaluated transportation and centralization, and highlighted that contemporary NiTi systems, such as ProTaper Next^®^, ProTaper Gold^®^, and XP-Endo Shaper^®^, are commonly investigated with respect to their ability to maintain canal anatomy and limit undesired deviations.

CBCT emerged as a widely adopted tool for three-dimensional assessment of root canal morphology before and after instrumentation, supporting non-destructive visualization of shaping outcomes in ex vivo research. However, important limitations related to spatial resolution and methodological variability across studies restrict the possibility of drawing firm comparative conclusions between specific systems or between manual and rotary techniques. Within these constraints, the available evidence suggests that both manual and rotary approaches can achieve acceptable shaping under controlled experimental conditions, but does not allow robust ranking of their relative clinical performance.

Overall, this review underscores that current research is clustered around a limited number of popular rotary systems and specific tooth types, while many instruments and clinical scenarios remain underrepresented. Future studies should prioritize more standardized protocols, broader inclusion of different tooth anatomies and instrumentation systems, and, where appropriate, higher-resolution imaging methods and clinical outcome measures. Such work will be essential to move from descriptive mapping of CBCT-based shaping studies toward stronger evidence to inform endodontic clinical practice.

## Figures and Tables

**Figure 4:**
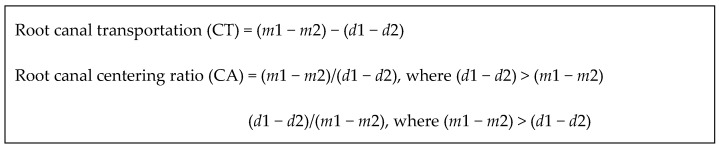
Formulas for determining the transportation and centralization of files in the root canal.

**Table 1:** Characteristics of the 28 studies included in this scoping review.

Author/Date	Teeth	Number of Teeth	Instrumentation Technique	Types of Files Used	Evaluation Mode	Results	Conclusions
**Dadresanfar et al., 2017** [24]	Maxillary first molars with a degree of curvature between 25 and 40°, extracted due to periodontal problems	32	Rotary Technique	iRace, and Reciproc	CBCT pre- and post-preparation	No significant differences were found between Reciproc and iRace regarding centralization or transportability after root canal preparation.	Both systems appear to be safe for preparing curved root canals.
**Karkehabadi et al., 2021** [25]	Mandibular first molars with mature apices and apical curvature of 10° to 30°	44	Rotary Technique	ProTaper and XP-Endo Shaper	CBCT pre- and post-preparation	ProTaper Universal causes greater canal transport in the buccolingual and mesiodistal directions than the XP-endo Shaper.	XP-Endo Shaper better preserves the original shape of the channel.
**Moura-Netto et al., 2015** [26]	Lower incisors extracted for periodontal reasons	50	Rotary Technique	Reciproc, WaveOne, and Tilos	CBCT pre- and post-preparation	There was similarity in the performance of the systems in planned areas, although the Tilos system presented a better standard of root canal preparation and a lower transport rate.	All systems tested had similar performance in terms of morphometric changes in flat areas, although the Tilos system presented a more anatomical preparation pattern and a significantly lower transport index.
**Adel et al., 2022** [27]	Lower and upper molars, extracted for periodontal reasons or caries	100	Rotary Technique	NeoNiTi, ProTaper, and Reciproc	CBCT pre- and post-preparation	Root canal transport in the NeoNiTi group was lower than in the other groups and significantly different from the ProTaper group (*p* < 0.05). There was remaining gutta-percha after retreatment in all four groups, which was not statistically significant (*p* > 0.05).	Although the NeoNiTi file produced less carryover than other file systems evaluated in the retreatment of curved root canals, all files were very effective at clinically acceptable levels.
**Shenoi et al., 2017** [28]	Mesiobuccal canals of lower molars with curvature angle varying from 20° to 40°	30	Rotary Technique	V-Taper 2H, ProTaper Next (PN), and HyFlex CM	CBCT pre- and post-preparation	All instruments maintained the original canal curvature with significant differences between the different files. The data suggested that the V-Taper 2H files presented better results for both variables evaluated. The V-Taper 2H files caused less transport and remained better centered in the canal than the PN and HyFlex CM files. However, it was found that PN caused less transport at the apical level than HyFlex CM.	Canal preparation with V-Taper 2H showed less transport and better centralization capacity than PN and HyFlex CM.
**Jainaen et al., 2018** [29]	Mesial root canals of extracted permanent molars with curvatures varying between 25° and 45°	40	Rotary Technique	WaveOne, Reciproc, ProTaper, and Mtwo	CBCT pre- and post-preparation	In the middle third of the canals, the reciprocating rotary files produced the smallest center deviation in the inner-to-outer furcal direction (*p* < 0.001). Transport was shown from the original channel shape in all directions in four groups. Reciprocating rotary files also took less preparation time (*p* < 0.001).	Reciprocating files result in less transportation and work time than rotational files.
**Elnaghy et al., 2016** [30]	Mesiobuccal canals of lower first molars with curvatures of 25–30°	40	Rotary Technique	ProTaper Gold and ProTaper Universal	CBCT pre- and post-preparation	There was no significant difference between the PG and PU systems in the average volume of dentin removed, canal transport, and centering ratio (*p* > 0.05).	The PG and PU NiTi rotary systems showed similar root canal shaping abilities in preparing mesial canals of mandibular first molars.
**Deepak et al., 2015** [31]	Mesiobuccal canals of lower first molars with 20–40° curvature	60	Rotary Technique	OneShape (OS), ProTaper Next (PTN), and Revo-S (RS)	CBCT pre- and post-preparation	The RS system maintained better channel centrality and lower transport compared to PTN and OS. There was no significant difference between the three groups in canal curvature after instrumentation.	All file systems are used to straighten the root canal curvature in a similar manner. RS instrumentation exhibited superior performance compared to OS and PTN systems with respect to transport and centering ratio.
**Wu and Zhu, 2014** [32]	Molars with canals with an angle of curvature ranging from 20° to 35°, which were extracted from patients who had periodontal and prosthetic problems	105	Rotary Technique–Crown-Down Technique was used	Reciproc, ProTaper SX, and Endomate DT	CBCT pre- and post-preparation	The results showed a statistically significant difference in root transport, found only in transverse sections 3.0 mm from the anatomical apex, between the “CR-500” group and the “CR-300” group. Furthermore, a significant difference was found in the centralization proportion between the “RM-300” group and the “CR-300” group. There was no significant difference in the two indices between the three groups at 1.5 mm and 6 mm cross-sections from the apex.	In the three situations in this study, the continuous rotation mode has better modeling capacity in root canal preparation than the RM mode when used with a single TF (size 25/0.06).
**Dhingra and Manchanda, 2014** [33]	Mandibular first molars extracted with curved mesial roots, curvature angles varying between 20 and 30°	100	Rotary Technique	PathFile (PF) and V-Glide Path 2	CBCT pre- and post-preparation	There was a statistical difference between the curvatures of the root canals and the working time between the two groups (*p* < 0.05). Canals were transported to the distal side in Group II, but there was slight mesial transport in Group I at 0 mm. Group I exhibited better centered capacity, except in the 1 mm range (*p* > 0.05). Changes in volume were statistically significant only in the 2 mm range (*p* < 0.05). The difference in cross-sectional area was not statistically significant at any interval (*p* > 0.05).	Nickel–titanium rotary files appeared to be suitable instruments for the safe and easy creation of sliding trajectories.
**Suzuki et al., 2022** [34]	Mandibular first or second molars extracted, with a root length of 16 mm, two separate and fully formed roots, a closed apex, and two mesial canals with independent foramina	54	Rotary Technique	WaveOne Gold, ProTaper Next, and ProTaper Universal	CBCT pre- and post-preparation		There was no significant difference between the groups when comparing the amount of filling material remaining after reinstrumentation (*p* > 0.05). The tested systems provided minimal change in root canal morphology in the apical portion after root canal retreatment. However, WOG promoted greater changes in root canal diameter.
**Pansheriya et al., 2018** [35]	Permanent teeth with mature root apices at 20–40°	30	Rotary Technique	ProTaper Next (PTN), SX Mani Silk system, and V-Taper SS White	CBCT pre- and post-preparation	The SX Mani Silk rotary system showed more channel carry and less centralized capacity than the PTN system.	The PTN rotary system has ideal centralized capacity and no canal transport compared to the SX Mani Silk and V-Taper SS White rotary file system.
**Öztürk et al., 2020** [36]	Single-rooted teeth extracted for periodontal reasons	72	Rotary Technique	XP-Endo Shaper and ProTaper Next	CBCT pre- and post-preparation	There were no statistically significant differences in PA, PO, and centralization ratio values between size 30 and size 35 instruments. Mean increases in PA and PO were statistically greater with XPS in size 40. PTNs had statistically greater buccolingual transport in sizes 30 and 35. XPS had lower mesiodistal transport values in the three apical sizes.	The PTN system is capable of removing dentin even in cases of increased apical diameter. However, XPS has less channel transport and better centralization ability compared to PTN.
**Pagliosa et al., 2015** [37]	Extracted human maxillary first molars were selected based on similar degrees of mesiobuccal canal curvature (20–40°) and radii (5–10 mm)	40	Rotary Technique	Hero 642 (HR), Liberator (LB), ProTaper (PT), and Twisted File (TF)	CBCT pre- and post-preparation	The results demonstrated no significant difference (*p* > 0.05) in the modeling ability between the rotational systems. The mean canal transport was: −0.049 ± 0.083 mm (RH); −0.004 ± 0.044 mm (LB); −0.003 ± 0.064 mm (PT); −0.021 ± 0.064 mm (TF). The average centralization capacity of the canal was: −0.093 ± 0.147 mm (RH); −0.001 ± 0.100 mm (LB); −0.002 ± 0.134 mm (PT); −0.033 ± 0.133 mm (TF). Furthermore, there was no significant difference between root segments (*p* > 0.05). It was concluded that the Hero 642, Liberator, ProTaper, and Twisted File rotary systems can be used safely in the instrumentation of curved canals, resulting in satisfactory preservation of the original canal shape.	The Hero 642, Liberator, ProTaper, and Twisted File rotary systems can be safely used in the instrumentation of curved canals, resulting in satisfactory preservation of the original canal shape.
**Saleh et al., 2019** [38]	Mesiobuccal root canals of mandibular molars with curvature angles varying from 25 to 40 degrees were randomly divided	32	Rotary Technique	Gentlefile (GF) and ProTaper Next (PTN)	CBCT pre- and post-preparation	The GF system produced more canal transport than the PTN system at 3 and 6 mm with a statistically significant difference (*p* ≤ 0.05), while at 9 mm, there was no statistically significant difference between the two systems (*p* > 0.05). PTN showed better centralization capacity than GF by 6 mm, and this difference was statistically significant (*p* ≤ 0.05). However, at 3 and 9 mm, there was no significant difference between the two systems (*p* > 0.05).	Under the limitations of this study, both systems produced canal transport, but the PTN system resulted in better root canal preparation with a lower degree of canal transport and better centralization ability than the GF system.
**Costa et al., 2017** [39]	Lower premolars, 18 mm, single long canal, oval in shape, and apical diameter ranging from 300 to 350 mm	45	Rotary Technique	ProTaper Universal, ProTaper Next, and Reciproc	CBCT pre- and post-preparation	All systems promoted extrusion of AT and apical debris; the latter was higher for the PTN group (*p* < 0.05). No system presented a perfect CA. The RC group demonstrated the highest DCRC (*p* < 0.05).	As a consequence of their use, large instruments promoted extrusion of TA and debris, regardless of the system used to perform the root canal preparation. Furthermore, no system has been able to remain perfectly centered within the root canal.
**Hazar et al., 2023** [40]	Mesiobuccal canals of mandibular molars	45	Rotary Technique	ProTaper Next, One Curve, and TruNatomy	CBCT pre- and post-preparation	No significant differences were observed between groups or root canal levels in either canal transport or centralization capacity (*p* > 0.05).	The TruNatomy system has demonstrated results comparable to the predecessor ProTaper Next and One Curve single-file systems.
**Capar et al., 2014** [41]	Mesiobuccal root canals of 120 mandibular first molars with curvature angles varying from 20° to 40°	120	Rotary Technique	OneShape, ProTaper Universal, ProTaper Next, Reciproc, Twisted File, and WaveOne	CBCT pre- and post-preparation	The R system removed a significantly greater amount of dentin than the OS, PU, and TFA systems (*p* < 0.05). There was no significant difference between the 6 groups in transport, canal curvature, changes in surface area, and centration ratio after instrumentation.	The 6 different file systems straightened the root canal curvature in a similar manner and produced similar canal transport in the preparation of mesial canals of mandibular molars. The R instrumentation exhibited superior performance compared to the OS, TFA, and PU systems with respect to volumetric change.
**Mamede-Neto et al., 2018** [42]	Mesiobuccal root canals of mandibular first and second molars	96	Rotary Technique	ProTaper Next, ProTaper Gold, Mtwo, BioRaCe, WaveOne Gold, and Reciproc	CBCT pre- and post-preparation	The highest mesiodistal (MD) transport (T) was found for Reciproc files (*p* < 0.05), and the highest buccolingual T (BL) for Reciproc, ProTaper Gold, and ProTaper Next files (*p* < 0.05). The highest Mesiodistal (MD) Centralization Capacity (CA) was found for BioRaCe files (*p* < 0.05), and the highest Buccolingual (BL) CA was found for BioRaCe and Mtwo files (*p* < 0.05).	All systems produced root canal transport. No file system has achieved perfect CA staging from scratch. Reciproc files had the highest MD T and BL T. BioRaCe files had the highest MD CA, while BL CA was similar for BioRaCe and Mtwo files.
**Al-Asadi et al., 2018** [43]	Permanent maxillary first molars with a range of mesiobuccal canal curvatures from 20 to 30 degrees	30	Rotary Technique	HyFlex EDM, Reciproc Blue, and OneShape	CBCT pre- and post-preparation	There were no significant differences in relation to canal transport, but there were significant differences in the apical third and no significant differences in the middle and coronal thirds in relation to the centralization ratio.	The three single rotary systems reported a degree in channel transport and centric ratio, but the HyFlex EDM reported the least.
**Mamede-Neto et al., 2017** [44]	Lower premolars	128	Rotary Technique and Manual Technique	WaveOne, WaveOne Gold, Reciproc, ProTaper Next, ProTaper Gold, Mtwo, BioRaCe, and RaCe	CBCT pre- and post-preparation	ProTaper Gold produced the lowest channel transport values, and RaCe the highest. ProTaper Gold files also had the highest centering capacity values, while BioRaCe files had the lowest. No significant differences were found between the different instruments in terms of channel transport and centering capacity.	All instruments used to prepare the root canal of mandibular premolars had similar performance with regard to canal transport and centralization capacity.
**Dhingra et al., 2015** [45]	Lower first molars were extracted due to periodontal problems	60	Rotary Technique	WaveOne and OneShape	CBCT pre- and post-preparation	The rotary system showed more effective wear in the danger zone when compared to alternative systems [24]. The distal area of the mesial root in lower molars is designated as the danger zone, making it a preferable location for strip drilling during instrumentation. On the other hand, the safety zone is the mesial region of the root, with a thicker layer of dentin that generally remains secured by endodontic instruments.	Reciprocating motion is better than rotational motion in all three parameters: canal transport, cross-sectional area, and cervical dentin thickness.
**Celikten et al., 2015** [46]	Upper first molars extracted with curvature of the mesiobuccal canal (25–35°)	50	Rotary Technique	ProTaper Next and New One Shape	CBCT pre- and post-preparation	Significant differences between apical and coronal levels were found for both systems (*p* < 0.05) in canal transport. When comparing the systems, similar values were found at each level, with no significant difference (*p* > 0.05) in terms of canal curvature and volume. Voxel sizes did not affect measurements of channel volume, curvature, or transport; no significant difference was found between voxel sizes of 0.100 and 0.125 mm^3^ (*p* > 0.05).	The ProTaper Next and New One Shape systems produced canal preparations with adequate geometry. The two voxel resolutions also showed similar results. Thus, the “best” voxel resolution would be 0.125 mm due to shorter scan time and reduced radiation exposure for in vivo studies.
**Mittal et al., 2017** [47]	Lower molars	20	Rotary Technique	Reciproc and OneShape	CBCT pre- and post-preparation	One shape and Reciproc had similar performances in terms of channel transport and centralization capacity.	The analysis revealed that Reciproc and OneShape did not present a statistically significant difference in terms of channel transport and centralization capacity (*p* > 0.05).
**Altufayli et al., 2022** [48]	Teeth with a curved root with angulation between 25 and 56 degrees	30	Rotary Technique	Reciproc Blue and One Curve	CBCT pre- and post-preparation	There was a significant difference in angle and radius of curvature (*p* < 0.05) after instrumentation for both One Curve and Reciproc blue groups, and no significant difference in change in working length after instrumentation of both One Curve and Reciproc blue groups (*p* > 0.05).	The blue Reciproc single-file system with reciprocating motion and One Curve with continuous motion causes a significant difference in the curvature and radius of the curved root canal, affecting the original shape of the root canal, without significant differences in the working length of the curved root canal.
**Damkoengsunthon et al., 2024** [49]	Mesial root canals of mandibular first molars	48	Rotary Technique	ProTaper Next (PTN), WaveOne Gold, and XP-Endo Rise Shaper (XPRS)	CBCT pre- and post-preparation	PTN removed more dentin and caused less RDT than XPRS. XPRS had less coronal transport. There were no differences in apical transport between systems.	XPRS performed best in DP, with less coronal transport and greater dentin preservation. All instruments showed good centration and minimal apical transport.
**Baabdullah et al., 2024** [50]	Root canals of artificial maxillary molars	60	Rotary Technique	Reciproc Blue	CBCT pre- and post-preparation	There was a significant difference in transport within the coronal and middle thirds. However, in the apical thirds, there were no significant differences. Both groups observed a significant difference in the centralization capacity in the coronal third.	Conservative access cavity may be recommended with caution as an alternative access to the traditional access cavity.
**Mehrabanian, et al., 2024** [51]			Rotary Technique	ProTaper Universal, ProTaper Gold, ProTaper Next, and WaveOne	CBCT pre- and post-preparation	The study compared the PTG, PTN, and WO systems, showing that each has strengths. PTG is more resistant and flexible, PTN removes less resin and centralizes better, and WO is simpler and more effective in the apical third, ideal for less experienced professionals.	The research highlights the importance of using data in choosing the ideal NiTi system for each clinical case, highlighting the role of microcomputed tomography in the objective evaluation of root canal preparation techniques. It is suggested that the choice of system considers the individual needs and experience of the professional.

## Data Availability

The original contributions presented in this study are included in the article. Further inquiries can be directed to the corresponding author.
